# Case report: Infective endocarditis caused by *Streptococcus sinensis*: The first case in mainland China and literature review

**DOI:** 10.3389/fcvm.2022.935725

**Published:** 2022-07-22

**Authors:** Yingmiao Zhang, Jing Wang, Yu Zhan, Ruizhi Tang, Hui Wang, Tian Qin, Zhongxin Lu

**Affiliations:** ^1^Department of Medical Laboratory, The Central Hospital of Wuhan, Tongji Medical College, Huazhong University of Science and Technology, Wuhan, China; ^2^State Key Laboratory for Infectious Disease Prevention and Control, Chinese Center for Disease Control and Prevention, Beijing, China; ^3^Cancer Research Institute of Wuhan, The Central Hospital of Wuhan, Tongji Medical College, Huazhong University of Science and Technology, Wuhan, China

**Keywords:** *Streptococcus sinensis*, infective endocarditis, emerging pathogen, 16S rRNA sequence, case report

## Abstract

*Streptococcus sinensis* was originally described as a causative agent for infective endocarditis in three Chinese patients from Hong Kong in 2002. Subsequently, several cases were reported outside Hong Kong, indicating that it is an emerging pathogen worldwide. We isolated a closely related strain in a young patient diagnosed with infective endocarditis in mainland China. In this paper, we reviewed the course of infection and provided a comprehensive comparison of its clinical characteristics with the reported cases.

## Introduction

Infective endocarditis (IE) is a potentially fatal disease that occurs on the endocardial surface of the heart, usually involving the heart valves. The viridans group streptococci is second only to *Staphylococcus aureus* as a causative agent of IE, which presents in ~20% of cases worldwide (1). Among these streptococcal species, *Streptococcus sinensis* (*S. sinensis*) was reported as a new pathogen isolated from a 42-year-old Chinese woman in Hong Kong with mitral regurgitation due to chronic rheumatic heart disease and IE in 2002 (2). Subsequent studies by Woo et al. found another two strains screened from 302 patients with bacteremia caused by viridans streptococci over a 6-year period, and demonstrated that *S. sinensis* is the common ancestor of the anginosus and mitis groups of streptococci (3, 4). In 2008, using the 16S rRNA sequencing method, the same group concluded that the oral cavity is the natural reservoir of *S. sinensis* (5). The increasing number of cases reported outside Hong Kong indicates that the organism is an emerging pathogen that is of interest globally (6–9).

## Case presentation

**Figure 1:**
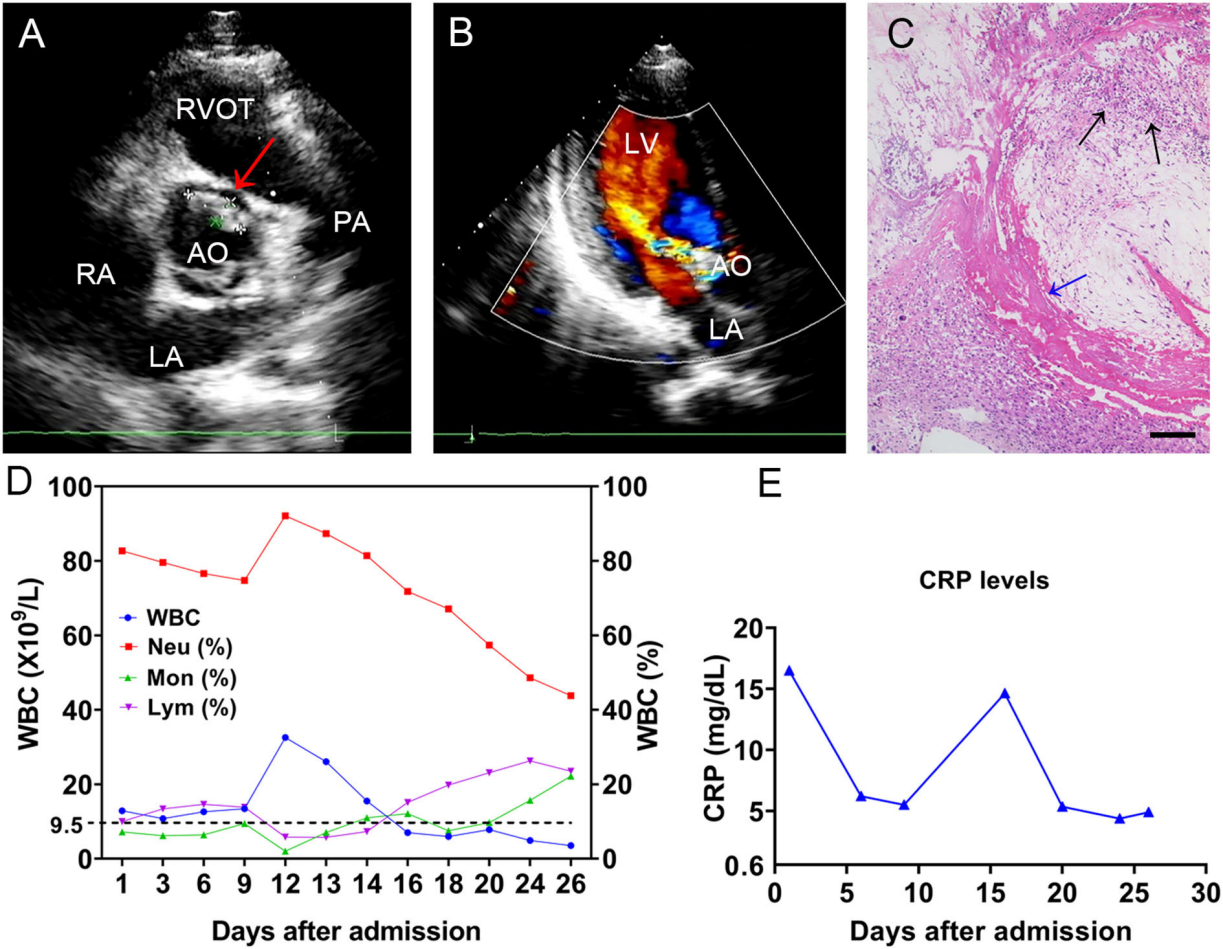
The TEE results of the mitral valve and WBC levels. TTE revealed **(A)** flocculent hypoechoic vegetations of the aortic valve (the red arrow) and **(B)** a moderate regurgitation bundle on the left ventricular outflow tract side during diastole of the aortic valve. **(C)** The histochemical analysis of the valve tissue revealed local myxoid and hyaline degeneration, missing endothelial cells, necrotic attached substances (the blue arrow), and neutrophil aggregation (the black arrows). Scale bars approximate 50 μm in length. The **(D)** WBC count and cell proportions and **(E)** CRP throughout the course. TTE, transthoracic echocardiography; LA, left atrium; LV, left ventricle; RA, right atrium; RVOT, right ventricular outflow tract; AO, aorta; PA, pulmonary artery; WBC, white blood cell; CRP, C-reactive protein.

A 19-year-old man with no obvious incentive for intermittent joint pain in both knees and the right elbow over the previous 2 months was admitted to our hospital on December 26, 2020. At admission, the patient complained of occasional numbness and pain in both feet, difficulty squatting, and lower extremity edema. Physical examination showed a sick male, with a blood pressure of 135/85 mmHg, a temperature of 38.1°C, a pulse of 110 beats/min, and a respiratory rate of 19 breaths/min. Laboratory tests revealed the following: a white blood cell (WBC) count of 12.9 × 10^9^/L (83.9% neutrophils), an erythrocyte sedimentation rate of 114 mm/h (normal, 0–20 mm/h), hypersensitive C-reactive protein (CRP, whole blood) of 16.5 mg/dl (normal, 0–0.8 mg/dl), hemoglobin of 87 g/L (normal, 120–160 g/L), total protein of 81 g/L (normal, 65–85 g/L), albumin of 25.9 g/L (normal, 40–55 g/L), and rheumatoid factor (RF) of 68.3 IU/ml. Urinalysis revealed a red blood cell count of 2,442/μl with heterogeneous morphology, urine protein of 2+, and a WBC count of 188/μl. Two sets of blood cultures were prepared before empirical antibiotic treatment with intravenous (i.v.) teicoplanin 1 g/day (qd) and cefoselis 2 × 1 g/day (q 12 h). Over the cardiac apex, a grade 2/6 proto-mesosystolic murmur was audible. Transthoracic echocardiography (TTE) revealed aortic valve bicuspid malformation (Type 0), flocculent hypoechoic vegetations of the aortic valve and anterior mitral valve leaflets (Figure 1A), and moderate regurgitation bundles on the left ventricular outflow tract side during diastole of the aortic valve (Figure 1B), supporting the diagnosis of IE. In all blood culture bottles, Gram-positive cocci were observed. The strain named BC1012612 was identified as *S. sinensis* by matrix-assisted laser desorption ionization/time of flight mass spectrometry (MALDI-TOF MS, Bruker Daltonik GmbH, Germany) with a high confidence level. A definite diagnosis of IE requires two major, one major with three minor, or five minor criteria (10). Thus, 6 days after admission, IE was diagnosed by the presence of two major and two minor criteria according to the modified Duke criteria (10).

**Figure 2:**
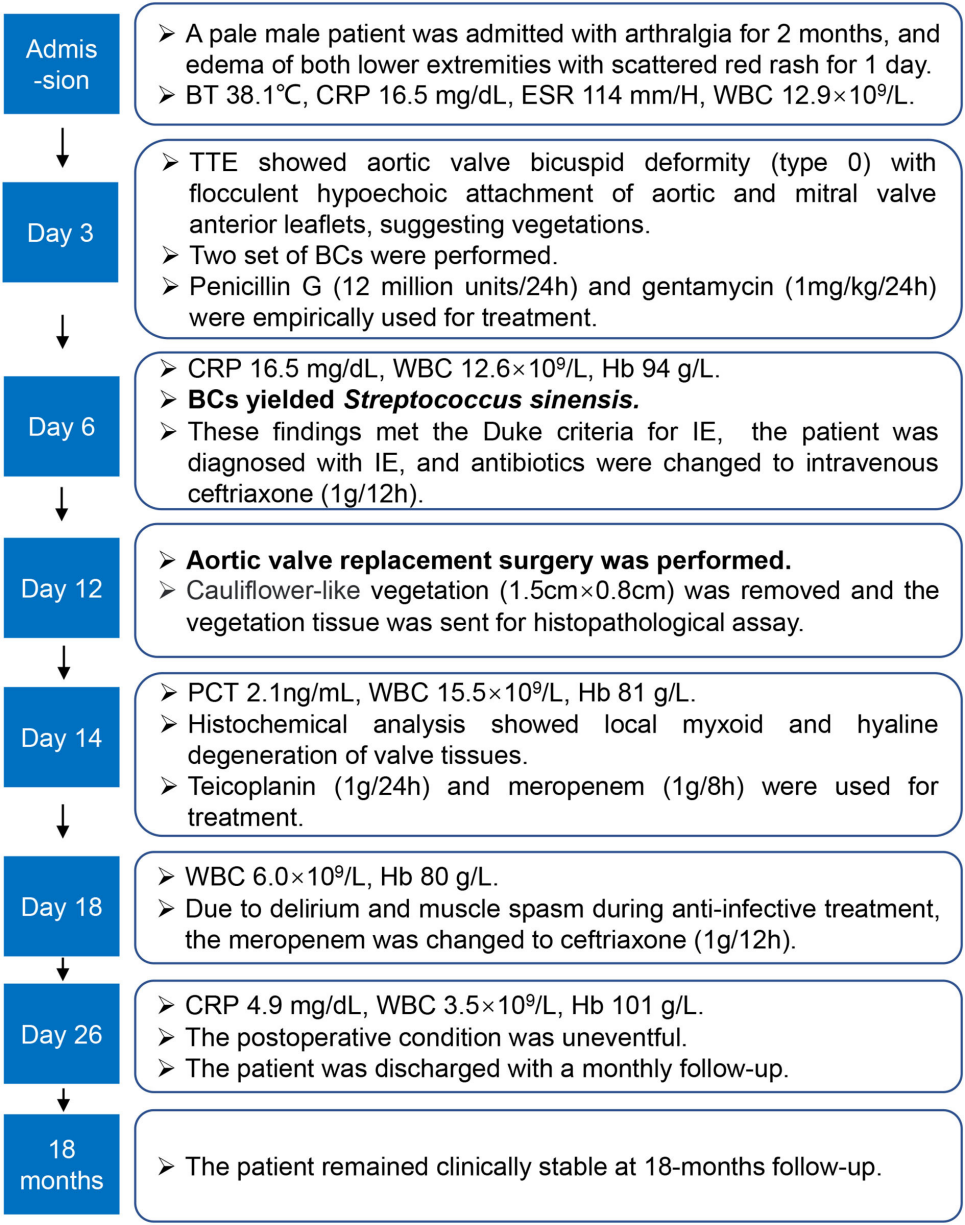
The timeline of the case presentation. The patient was discharged with a monthly follow-up 26 days after his admission. BT, body temperature; ESR, erythrocyte sedimentation rate; BCs, blood cultures; IE, infective endocarditis; PCT, procalcitonin.

An antibiotic susceptibility test *via* the Kirby–Bauer method showed the strain was susceptible to levofloxacin (32 mm), ceftriaxone (32 mm), linezolid (31 mm), vancomycin (23 mm), cefepime (32 mm), and chloramphenicol (28 mm). In addition, the minimal inhibitory concentrations (MICs) for penicillin G (0.064 mg/L) and meropenem (0.048 mg/L) were tested *via* an E-test. Based on the drug susceptibility result, the antimicrobial drug was changed to ceftriaxone (2 g/day). The patient underwent mechanical aortic valve replacement and mitral valve repair with no postoperative complications on Day 6 after his diagnosis. Histochemical analysis of the valve tissue showed local myxoid and hyaline degeneration, missing endothelial cells, and a large number of necrotic attached substances, and there were many neutrophils in the valve wall at the bottom of the necrotic substances (Figure 1C). The WBC count (Figure 1D) and CRP (Figure 1E) remained at high levels post-operation, and intravenous teicoplanin (1 g/day) and meropenem (1 g/8 h) were used for treatment. Due to the occurrence of delirium and muscle spasms during anti-infective therapy, meropenem was changed to ceftriaxone. After 2 weeks of symptomatic and supportive treatment, the patient's physiological conditions and inflammatory indicators were normalized, and he was discharged with a monthly follow-up 26 days after admission. At the time of writing, the patient has remained clinically stable for more than 18 months. Additionally, we present a timeline for the case presentation (Figure 2).

**Figure 3:**
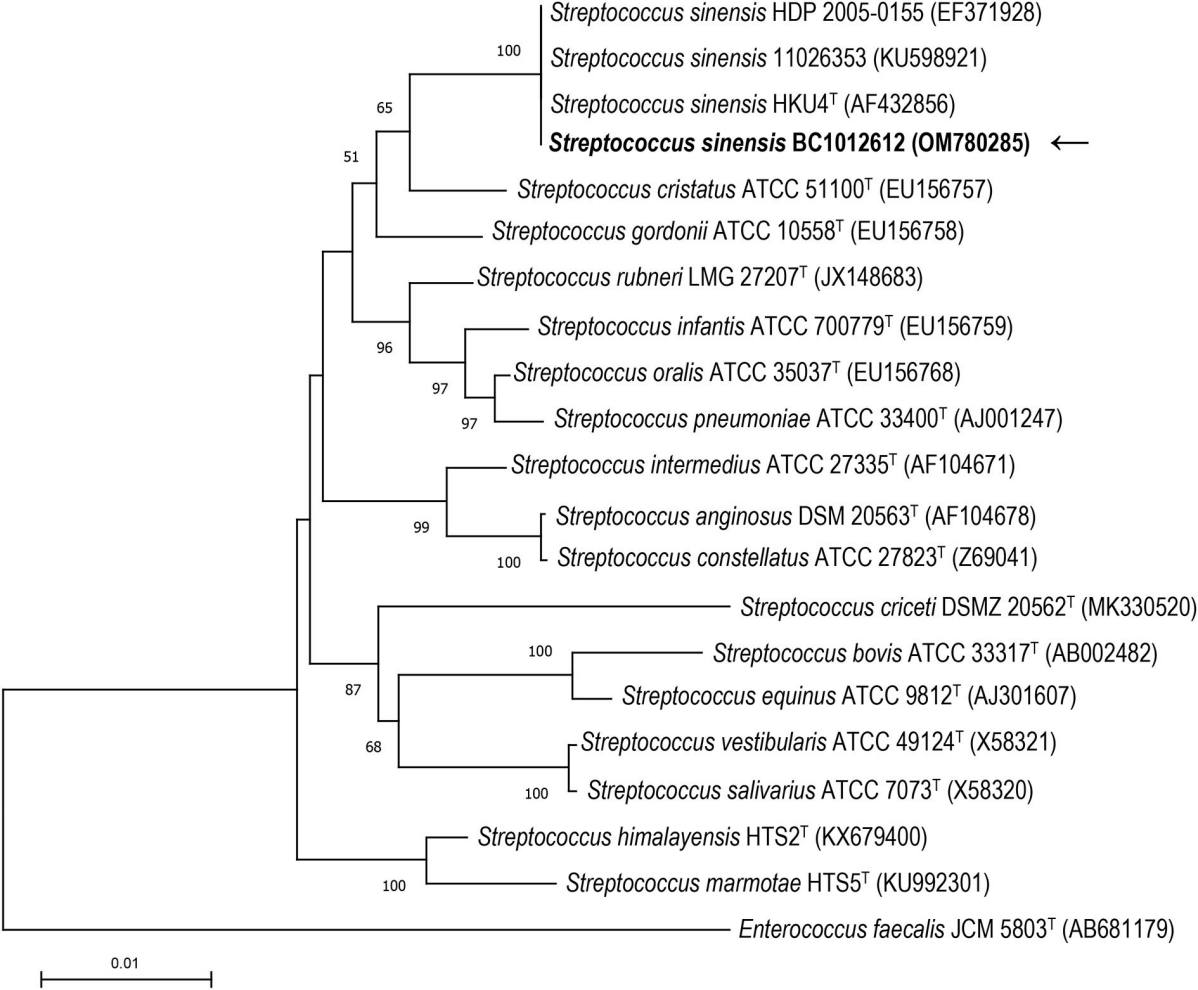
The phylogenetic tree based on the 16S rRNA gene sequences showing the relationship of isolated strain BC1012612 (the black arrow) and members within the genus *Streptococcus*. The tree was reconstructed by the neighbor-joining method, and *Enterococcus faecalis* JCM5803T was used as an outgroup. Bootstrap values (>50%) based on 1,000 replicates are shown at branch nodes. T, type strain.

16S rRNA gene sequence analysis was conducted to classify the isolated strain BC1012612 with universal 16S rRNA primers (forward primer: 5′-AGTTTGATCMTGGCTCAG-3′, reverse primer: 5′-GGTTACCTTGTTACGACTT-3′). A total of 1,439 contiguous nucleotides were determined. The complete 16S rRNA sequence was analyzed with the Basic Local Alignment Search Tool (BLAST) at the GenBank Database (https://blast.ncbi.nlm.nih.gov). The strain BC1012612 exhibited the highest (99.93%) 16S rRNA gene sequence similarity with the type strain of *S. sinensis* HKU4^T^ (GenBank accession No. AF432856). Among the 1,439 bases, there was only one base difference from strain HKU4^T^. Multiple alignments with sequences of the most closely related streptococci and the calculations of the levels of sequence similarity were carried out using CLUSTALW (11). A phylogenetic tree was constructed using the neighbor-joining method by using MEGA software version 11 (12). The topology of the phylogenetic tree was evaluated by using the bootstrap resampling method of Felsenstein (13) with 1,000 replicates. The phylogenetic tree showed that strain BC1012612 was clustered with strains HKU4^T^, HDP 2005-0155, and 11026353, and this cluster was strongly supported with a bootstrap value of 100% (Figure 3). The results of the comparative 16S rRNA gene sequence analysis demonstrated that the isolated strain BC1012612 belongs to the *S. sinensis* species. We have submitted the 16S rRNA sequencing results to GenBank (accession No. OM780285).

## Literature review and discussion

*S. sinensis*, a newly described species of viridans streptococci, was originally isolated in 2002 from blood cultures of a female patient with chronic rheumatic heart disease in Hong Kong (2). It has subsequently been reported in a few case reports outside Hong Kong, such as in Switzerland, France, Great Britain, and the Netherlands (6, 8, 9, 14–16), highlighting its importance as an emerging pathogen in the healthcare field. Continuous studies by Woo et al. revealed that the oral cavity is the natural reservoir of *S. sinensis* in healthy individuals, and that *S. sinensis* may be the common ancestor of the anginosus and mitis groups of streptococci according to clinical, phenotypic, and genotypic comparisons of these strains (4, 5). Based on phylogenomic and MALDI-TOF MS analysis, they proposed a new group called the “*sinensis* group,” which includes *S. sinensis, Streptococcus oligofermentans* (*S. oligofermentans*), and *Streptococcus cristatus* (*S. cristatus*), in 2014 (7). Jensen et al. reported *S. oligofermentans* as a later synonym of *S. cristatus* (17). With the development of genome sequencing technology, increasingly more details of *S. sinensis* have been explored.

IE caused by *S. sinensis* was diagnosed by blood cultures collected before antibiotic therapy. We isolated a strain of *S. sinensis* BC1012612 from the blood of a young patient with IE. The strain was identified by MALDI-TOF MS, and 16S rRNA sequencing was performed for further analysis. Among a total of 1,439 bases, the 16S rRNA sequence of this strain was found to have only one base difference from the type strain HKU4. A phylogenetic tree also showed a cluster within the previously reported *S. sinensis* strains and our strain BC1012612, suggesting that our strain is most closely related to the type strain. According to the emended taxonomy of the Mitis group of the genus *Streptococcus* carried out by Jensen et al., *S. sinensis*, together with *S. cristatus*, belongs to the *S. cristatus* clade but is distantly related to other strains and may represent a distinct taxon at the species level (17). The 16S rRNA sequencing method alone is inadequate for bacterial classification, and multidimensional analysis will be helpful for the accurate classification of the large numbers of species within genus *Streptococcus*.

The extant literature on *S. sinensis*-related IE is presented in Table 1. Unfortunately, a small number of cases have been reported without any clinical descriptions, making it impossible for a comprehensive summary and analysis of these cases (6, 18). Among the eight listed observations, including ours, there were only two female patients, and the age of the patients ranged from 19 to 63; the patient in our case is the youngest reported yet. All the patients had acquired congenital heart disease, and six out of eight patients were found to have vegetations and varying degrees of mitral regurgitation *via* echocardiography. It is intriguing that at least three patients with IE caused by *S. sinensis* had tooth problems, which may corroborate the oral origin of this organism. *S. sinensis* was also identified by a multiple PCR method in the subgingival plaque of two subjects with localized severe chronic periodontitis (19). However, more evidence is needed to ascertain whether *S. sinensis* is present in the oral cavity of individuals living in other regions outside Hong Kong. Two patients were reported to have travailed to Hong Kong or had undergone dental procedures there, thus hinting at the geographical reservoirs for *S. sinensis*. In addition, studies of the stomach and gut microbiota also revealed the presence of *S. sinensis* from gastric mucosa and stool samples at the genetic level *via* 16S rRNA sequencing (20, 21), but without any isolated strains. To date, no infections other than IE caused by *S. sinensis* have been reported in humans, suggesting that this organism may possess specific virulence factors to invade heart valves and cause damage. The sequence analysis of the manganese-dependent superoxide dismutase gene (sodA) *via* a PCR assay based on degenerate primers was initially carried out by Poyart et al. to identify the genus *Streptococcus* to a species level (22). A high congruence of strain grouping by MALDI-TOF MS in comparison with sodA sequence analysis regarding *streptococcus bovis/equinus*-complex was observed by Hinse et al., demonstrating the accuracy and reliability of MALDI-TOF MS in comparison to the DNA sequence-based method (23). It is difficult to identify viridans streptococci by traditional biomedical methods, even MALDI-TOF MS; genetic characterization should be performed to distinguish strains isolated in infectious diseases and the epidemiology of each species.

**Table 1:** Cases reported in *Streptococcus sinensis-*related IE.

**Strains**	**Gender**	**Age**	**Place**	**Asia** **origin**	**Vegetation**	**Antibiotics**	**Antibiotic duration^a^**	**Mitral involvement**	**Surgery**	**Follow-up**	**Years**	**References**
HKU4^T^	F	42	Hong Kong	Yes	No	AMP, CN	6 weeks	No	NA	NA	2000	(2)
Geneva	M	57	Switzerland	No	Yes	P, CN	6 weeks	Yes	Yes	Survival	1998	(6)
HDP2005-0155	M	55	France	No	No	AML, CN	8 weeks	Yes	Yes	Survival	2005	(14)
NA	F	20	France	No	Yes	AML, CN	4 weeks	No	Yes	Survival	2015	(15)
NA	M	63	Britain	Travel	Yes	AML,CN CRO	6 weeks	No	Yes	Survival	2019	(8)
11026353	M	37	France	No	Yes	AMC, CN	4 weeks	Yes	Yes	NA	2019	(16)
NA	M	58	Netherlands	Yes	Yes	P, CN	1 week	Yes	No	Death	2020	(9)
BC1012612	M	19	Mainland China	Yes	Yes	P, CN,CRO, MEM	6 weeks	Yes	Yes	Survival	2020	This study

a *Antibiotic duration includes the duration of treatment before and after surgery. NA, not available; F, female; M, male; AMP, ampicillin; CN, gentamycin; P, penicillin G; AML, amoxicillin; CRO, ceftriaxone; AMC, amoxicillin-clavulanic acid; MEM, meropenem*.

All previously reported patients were treated with ampicillin or amoxicillin, or with combined therapy with gentamicin. It seems that the strain is susceptible to the majority of antibiotics tested, especially β-lactam antibiotics, so the routinely used antibiotics could achieve an antibacterial effect. However, more antibiotics were applied in this case, such as ceftriaxone and teicoplanin, mainly due to the persistently high level of inflammatory markers during anti-infective therapy. Almost all the patients received mitral valve replacement because of the severity of the preexisting mitral regurgitation, and had a good outcome. The patient in this case underwent mechanical aortic valve replacement and mitral valve repair. Surgical exploration showed scattered and small vegetations on the left ventricular surface of the anterior mitral valve without obvious valve leaf thickening, ulceration, or involvement of chordae tendineae, so mitral valve repair was performed. Several studies have shown that mitral valve repair has low mortality, fewer complications, and a better long-term prognosis than mitral valve replacement (24–26). Of these cases, only one death occurred in a 58-year-old man who refused surgery and died of multiple cerebral infarctions several days after admission, suggesting that timely treatment is of great importance in the acute phase of infection.

## Conclusion

Herein, we report the first case of IE caused by *S. sinensis* in mainland China. The patient in our case is the youngest ever reported, and his initial symptoms were mainly joint pain. The patient was diagnosed with IE after the isolation of *S. sinensis* from blood cultures and treated with drug-sensitive antibiotics as well as surgery. The early identification of *S. sinensis* is critical to the diagnosis and treatment of IE, but routine biochemical methods and MALDI-TOF MS are not sufficient because of the variety of species within genus *Streptococcus*. Certain genetic methods, such as 16S rRNA sequencing, are required for the accurate classification of those obtained strains. In addition, the patients' travel history should be taken into consideration when determining the possible geographical reservoirs for this agent, as it has been proposed to be an oral flora present in Hong Kong. Our case highlights the importance of *S. sinensis* as an emerging pathogen and provides a comprehensive understanding of *S. sinensis*-related IE.

## Data availability statement

The datasets for this article are not publicly available due to concerns regarding participant/patient anonymity. Requests to access the datasets should be directed to the corresponding author.

## Ethics statement

The studies involving human participants were reviewed and approved by Medical Ethics Committee of The Central Hospital of Wuhan, Tongji Medical College, Huazhong University of Sciences and Technology. The patients/participants provided their written informed consent to participate in this study.

## Author contributions

Designed and conceived the experiments: ZL, HW, and YZhang. Performed the experiments: YZhang, JW, and YZhan. Analyzed the data: YZhang and RT. Wrote and reviewed the manuscript: YZhang, TQ, and ZL. All authors contributed to the article and approved the submitted version.

## Funding

This work was supported by a grant from the Wuhan Association for Science and Technology (Project No. HB2021C15) and by one local grant from The Central Hospital of Wuhan, Tongji Medical College, Huazhong University of Science and Technology to YZhang.

## Conflict of interest

The authors declare that the research was conducted in the absence of any commercial or financial relationships that could be construed as a potential conflict of interest.

## Publisher's note

All claims expressed in this article are solely those of the authors and do not necessarily represent those of their affiliated organizations, or those of the publisher, the editors and the reviewers. Any product that may be evaluated in this article, or claim that may be made by its manufacturer, is not guaranteed or endorsed by the publisher.
